# Evaluation of IL-17B and IL-17F mRNA expression in peripheral blood mononuclear cells and association with clinical outcome of IBD patients 

**Published:** 2017

**Authors:** Mohammad Taghi Safari, Vahid Chaleshi, Peyman Tarban, Mahyar Nourian, Hedieh Balaii, Shabnam Shahrokh, Hamid Asadzadeh Aghdaei

**Affiliations:** 1 *Gastroenterology and Liver Diseases Research Center, Research Institute for Gastroenterology and Liver Diseases, Shahid Beheshti University of Medical Sciences, Tehran, Iran *; 2 *Basic and Molecular Epidemiology of Gastrointestinal Disorders Research Center, Research Institute for Gastroenterology and Liver Diseases, Shahid Beheshti University of Medical Sciences, Tehran, Iran*

**Keywords:** Inflammatory bowel disease, Crohn’s disease, Ulcerative colitis, Interleukin-17, qPCR

## Abstract

**Aim:**

In this study, we determined the gene expression analysis of IL-17 gene family for early detection of subclinical inflammation among IBD patients.

**Background:**

Cytokines have a vital role in the pathogenesis of inflammatory bowel disease (IBD). Interleukin-17 is the signature cytokine of the recently identified T helper 17 (Th17) cell subset. IL-17F is mainly involved in mucosal host defense mechanisms whereas the functions of IL-17B remain largely elusive.

**Methods:**

In this cross-sectional study, IBD patients divided into two active and inactive groups. Peripheral blood mononuclear cells (PBMCs) from 38 IBD patients which 20 inactive samples and 18 active individuals were collected. Changes of IL-17 F and IL-17B mRNA expression level evaluated by quantitative-real time-PCR.

**Results:**

mRNA expression level of IL-17B and IL-17F in CD, UC, active and inactive groups have been assessed and there were no significant differences (P>0.05). Patients were classified into five different categories as follows: i) 5ASA; ii) 5ASA + Pred; iii) 5ASA + AZA; iv) 5ASA + Pred + AZA; v) 5ASA + Pred + AZA + IFX according to medication usage, expression of IL-17F and IL-17B had no differences (p>0.05).

**Conclusion:**

Evaluation of IL-17B and IL-17F mRNA expression level illustrate no difference among active and inactive patients. Therefore, IL-17B and IL-17F are not biomarkers in an Iranian IBD patients.

## Introduction

 Inflammatory bowel disease (IBD) is a multifactorial disorder that its symptoms include diarrhea with bleeding, fever and fatigue, Abdominal pain, reduced appetite, unintended weight loss in the gastrointestinal tract (1, 2). Although the exact cause of IBD still remains unknown, various factors such as genetics factors, environmental factors, microbial intestinal flora and immunologic component can be involved for manifesting of IBD (3). Ulcerative colitis (UC) and Crohn's disease (CD) are two most common IBD entities that characterized by the location of inflammation in GI (4). The rate of patients who suffer from IBD has increased in recent years. A number of studies have reported that more than three million patients in the United States and European countries discomfort of IBD, in IRAN the rate of this condition has significantly increased in the last decade (5, 6). The role of genetics in IBD has been supported by studies on family history and monozygotic twins (7, 8). In recent investigations, about 163 gene loci have been associated with IBD since 110 genes have related to both UC and CD, 30 specific genes are associated with CD and 23 genes are considered as specific for UC (9, 10). The role of IL-17 that is the 20-kDa glycoprotein in inflammatory disease as a pro-inflammatory have been indicated (8). IL-17 is secreted exclusively by active T helper cells. Indeed, the past studies assessing sequence screening about IL-17 illustrates six subgroups of IL-17, from A to F (9). IL-17F isoform 1 and 2 have the highest degree of homology with IL-17A among all subgroups of IL-17 and both have the vital role as a pro-inflammatory cytokine (8, 9). IL-17B is expressed in various organs like trachea, prostate, lung, small intestine, testes, adrenal, and pancreas (10). IL-17B and IL-17F can induce pro-inflammatory cytokines like tumor necrosis factor (TNF) (11). Recent studies have investigated the role of IL-17 in a number of diseases like Rheumatoid Arthritis as an autoimmune disease (12). This paper will focus on evaluating mRNA expression level of IL-17B and IL-17F in PBMC of Iranian population patients with IBD. In addition, mRNA expression level of IL-17B and IL-17F compared between active and inactive phases of IBD patients. 

## Methods

**Population**

In this cross-sectional study, 38 participant subjects (20 inactive, 18 active) who referred to Gastroenterology and Liver Diseases Research Center, Research Institute for Gastroenterology and Liver Diseases, Shahid Beheshti University of Medical Sciences, Tehran, Iran from 2013 to 2016. A questionnaire including demographic and clinical features filled for all patients. In addition, 6 ml whole blood collected in EDTA tubes and stored at 4˚C until RNA extraction. The consent form was obtained from patients that ethics committee at the Research Center Gastroenterology and Liver Diseases (RCGLD) approved the protocol. 

**Table 1:** Primers were used for quantification

Gene symbol	Primer sequence	TM (°C)	Product length	GC (%)	Length
*IL-17B*	F:5'- GAGCCCCAAAAGCAAGAGGAA-3' R:5'- TGCGGGCATACGGTTTCATC-3	60.82 61.09	107bp	52.38 55.00	21 20
*IL-17F*	F:5'- GCGTTTCCATGTCACGTAACA-3' R:5'- CAGCCCAAGTTCCTACACTGG-3'	59.47 60.61	121bp	47.62 57.14	21 21
*B2M*	F:5'-TGCTGTCTCCATGTTTGATGTATCT-3' R:5'-TCTCTGCTCCCCACCTCTAAGT-3'	60.34 61.98	86bp	40.00 54.55	25 22

**Table 2:** Demographic characteristics of the study population

Variable		Inactive	Active	Total
BMI		23.5811±6.96749 (11.73-38.57)	28.6188±4.82197 (20.76-37.11)	25.7977±6.51902 (11.73-38.57)
Age of diagnosis		30.68±12.763 (17-55)	30.67±11.788 (15-51)	30.68±12.157 (15-55)
SEX	Male	7(35.0%)	7(38.9%)	14(36.8%)
	Female	13(65.0%)	11(61.1%)	24(63.2%)

**Figure 1:**
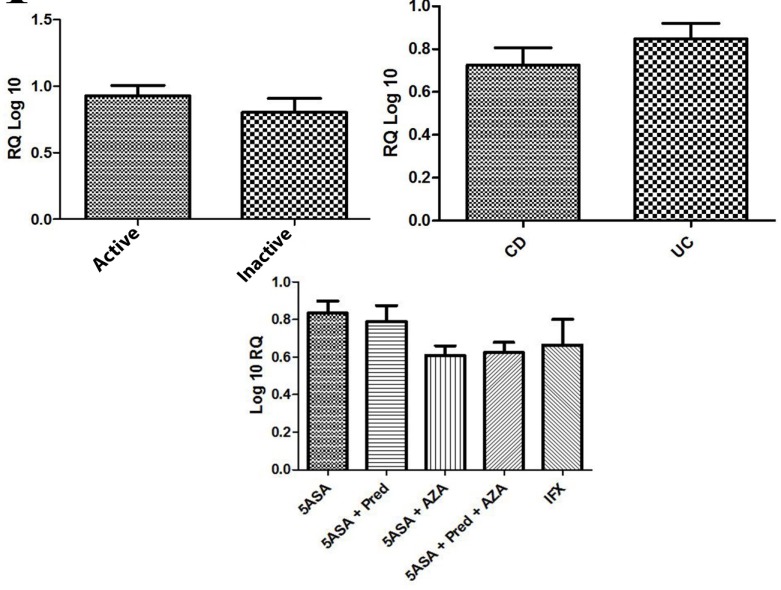
IL-17B mRNA expression level at different IBD pathological phases, according to the type of IBD (UC or CD) and according to drug history

**Figure 2:**
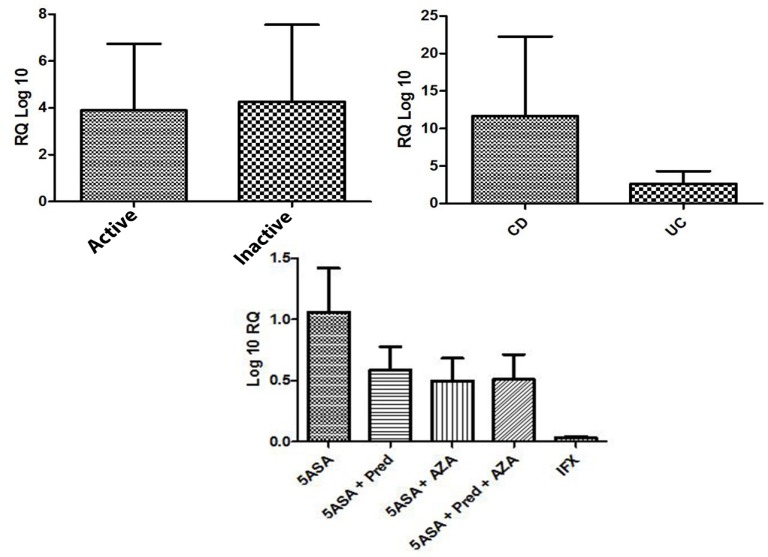
IL-17F mRNA expression level at different IBD pathological phases, according to the type of IBD (UC or CD) and according to drug history

**RNA Isolation and Real-Time**

Total RNA was extracted from PBMC and YTA RNA Extraction (YEKTA TAJHIZ AZMA) was utilized. Spectrophotometric optical density measurement (260 and 280 nm) was used for evaluation of quality and quantity of RNA. cDNA synthesis used by Revertaid RT Reverse Transcription Kit (Thermo Fisher Scientific, Catalog number: K1691). For evaluation of IL-17B and IL-17F expression ABI 7500 real-time (2.3 version) PCR system (Applied Biosystems, Foster City, CA, USA) were employed with Takara SYBR Master Mix instructions (Shiga, Japan). Primers for IL-17B, IL-17F, and Beta-2-Microglobulin (as a housekeeping gene) were designed by using Gene Runner and Primer3 online programs (Table 1). 

**Statistical Analysis**

Determination of gene expression by Student t-test was apprised. One-way ANOVA and Student t-test were used to examine the IL-17B and IL-17F mRNA expression level between groups. Graph pad prism 5 software (Graph Pad Software, Inc. La Jolla, CA, USA/ (https://www.graphpad.com/scientific-software/prism/) were utilized to draw the graphs. P<0.05 regarded as an indication of statistically significant difference. 

## Results

**Demographics**

Thirty-eight patients have participated in the present study. Accurately, 20 and 18 individuals were in inactive and active phase respectively. To be more specific, 20 patients were in inactive phase, including UC 77.3% and CD 22.7% and 18 patients were in active phase which all of them were UC. In both groups, the rate of the male was 36.8%, the percentage of the female was 63.2%, and there was no significant association between them. The average of patients’ diagnosis age and BMI were 30.68 (minimum 15, maximum 55 ± 12.157) and 25.7977(minimum 11.73, maximum 38.57± 6.5) respectively (Table 2).

**Evaluation of IL-17B and IL-17F**

mRNA expression level of IL-17B and IL-17F in CD, UC, active and inactive groups have been assessed and there were no significant differences (P>0.05). Additionally, an evaluation of patients who had a history of taking medication was also conducted and there was no association among this groups (P>0.05). Patients were classified into five different categories as follows: i) 5ASA; ii) 5ASA + Pred; iii) 5ASA + AZA; iv) 5ASA + Pred + AZA; v) 5ASA + Pred + AZA + IFX according to medication usage (Figure II and I).

## Discussion

Revolution of the immune system in IBD patients has been investigated in many studies. It is addressed that impaired mucosal cytokine secretion has a direct effect on pathogenesis of IBD (11, 12). The presented study aimed to evaluate mRNA expression level of IL-17B and IL-17F in PBMC, which is the non-invasive and exclusive for patients of active and inactive IBD patients. Although the mRNA expression level of IL-17B was higher than IL-17F in subjects, there was no significant association between expression of both genes and IBD. IL-17 as a pro-inflammatory secretor, mainly derived from Th-17 (13). IL-17 as a mediator producing cells can facilitate the development of inflammation and colorectal carcinoma by promoting angiogenesis and production of VEGF (vascular endothelial growth factor) by tumor cells. In addition, the modulation response of IL17F may inhibit tumor angiogenesis and enhance the inflammatory response of the host to tumorigenesis (14, 15). Investigating on various subgroups of IL-17 has been done in IBD. For instance, in 2014 study of IL-17C in IBD patients in Germany by M Friedrich et.al, illustrated the increase in IL-17C mRNA serum level of patients (16). In 2008 Holtta Veera et al. evaluated mRNA expression level of cytokines such as IL-17, IL-23, TNF-a, etc in biopsy and stool of Crohn’s patients. Their results illustrate association in IL-17 and IL-23 connected to the etiology of patients (17). Study mRNA expression level of IL-12 and IL-17 which conducted by Nielson and colleagues in biopsy of IBD patients in 2003 illustrated association of these genes in IBD. Their study suggested, these two genes might be a therapeutic target in future for IBD (18). In 2007, Julia Seiderer studied mRNA expression level and p His161Arg polymorphism of IL-17F gene in biopsy of German IBD patients. Their results showed that association of mRNA expression level of IL-17F by Crohn’s patients (19). According to our knowledge, evaluation of IL-17B and IL-17F mRNA expression in peripheral blood mononuclear cell (PBMC) was investigated for the first time in our study (17, 19). Indeed, several previously studies have shown significant results that is different from our results. The reason for these contradictory findings is not clear. Ethnic heterogeneity, sample type, gene environment interactions and different sample size may be the probable explanation for this discrepancy. Finally, we could not find any evidence to support an association between IL-17B and IL-17F mRNA expression in peripheral blood mononuclear cell (PBMC) and IBD patients. Therefore, in future studies, we suggest that cellular mechanisms and other sub-units of IL-17 be investigated.

The present study examined association of IL-17B and IL-17F mRNA expression level illustrate no difference among active and inactive patients. In addition, expression of IL-17F and IL-17B had no differences (p>0.05) in patients who had a history of taking the four types of drug (5-ASA, Pred, AZA or IFX). Thus, these analyses may be used in the prognosis, diagnosis and treatment of IBD.
